# Safety assessment of compliant, highly invasive, lipid A-altered, O-antigen-defected *Salmonella* strains as prospective vaccine delivery systems

**DOI:** 10.1186/s13567-022-01096-z

**Published:** 2022-10-01

**Authors:** Ram Prasad Aganja, Chandran Sivasankar, Chamith Hewawaduge, John Hwa Lee

**Affiliations:** Department of Veterinary Public Health, College of Veterinary Medicine, Jeonbuk National University, Iksan, 54596 South Korea

**Keywords:** *Salmonella*, intracellular survival, delivery strain, safety, endotoxicity

## Abstract

In the present study, two prospective *Salmonella* delivery strains, JOL2782 and JOL2837, were developed by gene deletions of *lon* and *cpxR*, which are related to cellular adhesion and intracellular survival. Additionally, *sifA* deletion was introduced for JOL2782, which confers immune susceptibility and improves antigen delivery. Similarly, the *rfaL* deletion and *lpxE* substitution for *pagL* were accomplished in JOL2837 to reduce virulence and endotoxicity. Thus, enhanced adhesion and invasion and reduced intracellular survival were attained. Furthermore, aspartic acid auxotrophic (*asd*) was deleted to impose Darwinian selection on retention of the foreign antigen-expressing plasmid. Both delivery strains induced sufficient cytokine expression, but the level was significantly lower than that of the wild-type strain; the lowest cytokine expression was induced by the JOL2837 strain, indicating reduced endotoxicity. In parallel, IgG production was significantly enhanced by both delivery strains. Thus, the innate and adaptive immunogenicity of the strains was ensured. The environmental safety of these strains was ascertained through faecal dissemination assays. The nonpathogenicity of these strains to the host was confirmed by body weight monitoring, survival assays, and morphological and histological assessments of the vital organs. The in vitro assay in murine and human cell lines and in vivo safety assessments in mice suggest that these novel strains possess safety, invasiveness, and immunogenicity, making them ideal delivery strains. Overall, the results clearly showed that strain JOL2782 with *sifA* deletion had higher invasiveness, demonstrating superior vaccine deliverability, while JOL2837 with *lpxE* substitution for *pagL* and *rfaL* deletion had outstanding safety potential with drastically abridged endotoxicity.

**Supplementary Information:**

The online version contains supplementary material available at 10.1186/s13567-022-01096-z.

## Introduction

*Salmonella enterica* serovar Typhimurium (ST), a gram-negative intracellular pathogen, infects both animals and humans. ST is a promising organism for use as a live vaccine and drug vector due to its intrinsic properties [1, 2]. Veterinary vaccinations have played an important role in safeguarding animal and human health, minimizing animal illness, and facilitating efficient livestock production to support the rising human population. Vaccination of farm animals is challenging in terms of cost, immunization sustainability, and safety. For this issue, *Salmonella* is an intracellular bacterial pathogen with high invasive potential and stimulates strong mucosal, humoral and cellular immune responses. Genetically altered nonpathogenic *Salmonella* is an attractive element to develop as a delivery system for veterinary and human applications. Recent advancements in molecular and synthetic biology have facilitated the modification of ST as an attractive DNA delivery vehicle for a diverse range of payloads, such as immunogens, heterologous antigens, and therapeutic and antitumour drug molecules [3, 4]. Such a bacteria-mediated delivery system has the benefits of high scalability in a short span, cost-effectiveness, and high efficacy. A potential live attenuated vaccine and drug carrier strain demand efficient localization to express the recombinant protein and elicit an immune response without any significant systemic infiltration. The premier characteristics of an ideal bacterial delivery vector include sufficient attenuation, safety, and localization in the targeted region for active antigen presentation, along with stable maintenance of an antigen coding recombinant plasmid vector. Since salmonellosis is an emerging burden in the animal industry, it needs to be restrained in the host while using it as a delivery vector. For successful attenuation of ST, mutants deficient in the global regulatory system, biosynthetic precursors, and auxotrophic mutants have been studied [5–8].

*Salmonella* invasion is an intricate process primarily controlled by genes residing in *Salmonella* pathogenicity islands I (SPI-1) and II (SPI-2). The Lon protease is a global regulator of the expression of early virulence genes required for bacterial pathogenesis [9]. This molecule is a negative regulator of SPI-1, and its mutation impairs intracellular survival [10]. The CpxR protein promotes virulence, and the removal of *cpxR* enhances fimbriae expression on the cell surface, which preserves efficient adhesion and uptake by antigen-presenting cells [11, 12]. With this background, a live attenuated *Salmonella* strain for vaccine delivery has been developed from a wild-type *Salmonella* strain (JOL401) via deletion of the SPI-1 *lon* and *cpxR* genes [13]. The deletion of *sifA* from SPI-2 disrupted the integrity of *Salmonella*-containing vacuoles (SCVs) [14] and prevented escape from immune recognition. Thus, for release of a plasmid containing the target gene into the cytoplasm, the mutant strain *Salmonella* JOL2500 (*Δlon, ΔcpxR, ΔsifA*) with selective auxotrophy was developed as a vaccine vector system [15].

The limitation of live attenuated organisms for therapeutic applications is interference from pre-existing antibodies [16]. To address such differentiation of infected and vaccinated animals (DIVA) and to avoid pre-existing immunity conferred by O-antigen, researchers deleted O-antigen ligase (*rfaL*) from the ST genome [5]. Furthermore, the strain was equipped for minimum endotoxicity by replacing *pagL* (lipid A deacylase) with *lpxE* [17], an inner membrane phosphatase from *Francisella tularensis*, to produce 1-monophosphorylated lipopolysaccharide (MPL). This complementation of *pagL* with *lpxE* minimizes the endotoxicity of *Salmonella* but retains immunogenicity [17]. Hence, we engineered a live attenuated *Salmonella* JOL3000 vector system with deletions of *lon, cpxR, rfaL,* and *pagL::lpxE* from the ST genome [18]. Antibiotic selection cannot be implemented in antigen-expressing plasmids and delivery strains due to the risk of acquisition of drug resistance by the host-microbial consortia. Hence, as promising safe vaccine delivery systems, both JOL2500 and JOL3000 were engineered with aspartate semialdehyde dehydrogenase (*asd*) gene deletion for aspartic acid auxotrophy. Thus, Darwinian selective pressure was imposed on the vaccine delivery system to retain the antigen-expressing plasmid [19].

An ideal vaccine delivery strain should be less pathogenic, environmentally safe, and localize in the target region without unintended effects. Based on this, live attenuated strains for the payload of heterogonous antigens were developed. In this study, our focus was on the safety assessment of developed delivery strains with multiple gene deletions. To this end, the bacterial load in the organs and their morphological and histological assessment were considered. Faecal discharge of the delivery strains was analysed to ensure environmental safety. Regarding immunogenicity, adhesion, and invasion, IgG production and cytokine expression were studied. The novel combinations of ST gene deletions resulted in the development of nonpathogenic, efficacious *Salmonella* delivery strains.

## Materials and methods

### Bacterial strains, plasmids, and primers

**Figure 1:**
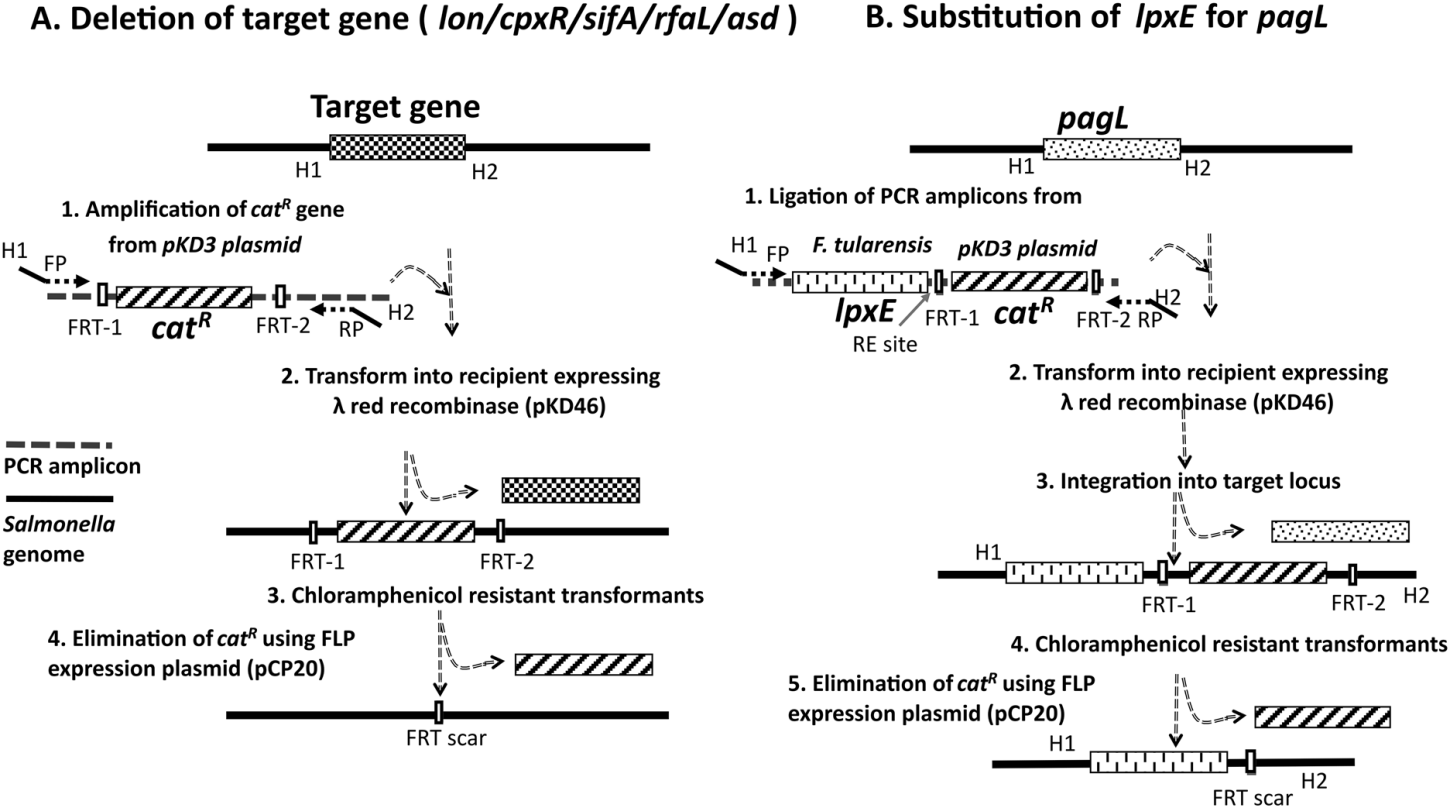
**Diagrammatic representation for deletion and substitution of genes using the λ red recombinase system.** **A**. Deletion of the target gene (*lon/cpxR/sifA/rfaL/asd*). *cat*^*R*^ was amplified from the pKD3 plasmid with the aid of primers designed by incorporating homologous regions from the target gene to be deleted. Bacterial strains were transformed with pKD46 to express λ red recombinase. Then, the amplified gene was transformed into pKD46 transformants, and the chloramphenicol-resistant transformants assured the deletion of the target gene. **B**. Substitution of *lpxE* for *pagL*. *lpxE* and *cat*^*R*^ were amplified and ligated. The ligated construct was amplified using primers designed based on upstream and downstream homologous regions of *pagL*. pKD46 transformant bacterial strains were electroporated with the amplified linear DNA. Screening in chloramphenicol-containing plates confirmed the replacement of *pagL*. *cat*^*R*^ was eliminated with the expression of pCP20. H1 and H2—homologous regions, FP, RP—forward and reverse primers, FRT-1/2—flippase recognition target, *cat*^*R*^—chloramphenicol acyl transferase.

The bacterial strains, plasmids, and primers used in this study are listed in Tables 1, 2. All bacterial strains were routinely grown in Luria Bertani (LB; BD, Sparks, MD, USA) medium at 37 °C. The parent mutant *Salmonella* Typhimurium strain JOL911 (Δ*lon* and Δ*cpxR*) [13] was engineered to develop *Salmonella* JOL2500 by deletion of *sifA,* and *Salmonella* JOL3000 was made by deletion of *rfaL* and *pagL* complemented by *lpxE* by employing the λ red recombination procedure as described elsewhere with modifications [20] (Figure 1). The *asd* gene was removed from both strains using the same procedure. This recombineering utilizes the insertion of an antibiotic resistance cassette into the chromosome in place of the target gene. Briefly, the parent ST strain was transformed with the helper plasmid pKD46 and induced with L-arabinose for recombinase expression. The linear DNA cassette of the *cat*^*R*^ gene flanked by a homologous sequence of the target gene was amplified from pKD3 and electroporated (Harvard Apparatus, USA) in pKD46-transformed *Salmonella* to delete the target gene. The target gene-deleted mutant colonies were selected by plating on LB broth containing 25 μg/mL chloramphenicol. *lpxE* from *F. tularensis* and *cat*^*R*^ were PCR-amplified and ligated for *pagL* substitution. The recipient strain was electroporated with the PCR-amplified linear DNA cassette. The chloramphenicol-screened colonies were selected to eliminate the FRT-flanked *cat*^*R*^ by transforming the pCP20 plasmid that encodes flippase. The attained mutants were confirmed by the respective flanking (outer) primers. The expression of the heterologous antigen in these strains was performed by cloning the open reading frame (ORF) of the gene encoding the HA globular head region from the H1N1 influenza virus into the pJHL204 plasmid using the AscI and PacI restriction sites. The cloned plasmid was then transformed into *Salmonella* JOL2500 and *Salmonella* JOL3000 and labelled *Salmonella* JOL2782 and *Salmonella* JOL2837, respectively.

**Table 1:** **List of bacterial strains and plasmids used in the present study**.

Bacteria/plasmid	Genotypic characteristics	References
*S*. Typhimurium		
JOL401	Wild-type (WT)	Lab stock
JOL911	Δ*lon*, Δ*cpxR*	[13]
JOL2500	Δ*lon*, Δ*cpxR*, Δ*sifA*, Δ*asd*	[15]
JOL3000	Δ*lon*, Δ*cpxR*, Δ*rfaL*, Δ*pagL*::*lpxE,*Δ*asd*	Lab stock
JOL2782	JOL2500 carrying pJHL204-H1N1 HA	In this study
JOL2837	JOL3000 carrying pJHL204-H1N1 HA	In this study
*E. coli*		
DH5α	*E. coli* F^−^Φ80dlacZΔM15Δ (lacZYA-argF) U169recA1 endA1 hsdR17(rk-mk^+^) phoA supE44 thi1 gyr A96 relA1λ-	Lab stock
BL21(DE3)	F^–^, ompT, hsdSB (rB^–^mB^–^), dcm, gal, λ(DE3)	Lab stock
* E. coli* 232	F—λ—φ80Δ(lacZYA-argF) endA1 recA1 hadR17 deoR thi-1 glnV44 gyrA96 relA1 ΔasdA4	Lab stock
* F. tularensis*	Source for *lpxE* amplification	Lab stock
Plasmid		
pET28a( +)	IPTG-inducible expression vector; Kanamycin resistance	Novagen
pJHL204	*asd* + , CMV promoter, SV40 promoter, pBR322 ori	Lab stock
pKD46	Ori101-repA101ts; encodes Lambda red genes (*exo*, *bet*, *gam*); native terminator (tL3); arabinose-inducible for expression (ParaB);*bla*	[20]
pKD3	oriR6K gamma, *bla* (ampR), *rgnB* (Ter), *cat*^*R*^, FRT	[20]
pCP20	Helper plasmid, contains a temperature-inducible *flp* gene for removing the FRT flanked chloramphenicol gene	[41]

**Table 2:** **List of primers used in this study**.

Gene	Primer	5'– 3' Sequences	References
TNF-α	Forward	CATCTTCTCAAAATTCGAGTGACAA	[42]
	Reverse	TGGGAGTAGACAAGGTACAACCC	
IFN-γ	Forward	TCAAGTGGCATAGATGTGGAAGAA	[42]
	Reverse	TGGCTCTGCAGGATTTTCATG	
IL-1β	Forward	TTCACCATGGAATCCGTGTC	This study
	Reverse	GTCTTGGCCGAGGACTAAGG	
*invF*	Forward	GCAGCAAATTATTACGCCTTC	[26]
	Reverse	AGTCTTCTCCCAGCATTCTC	
*ompF*	Forward	CGTGCTGGCGGTTTGTTGAC	[43]
	Reverse	TTGCTGTACGCTGCGGTGAC	
*β-actin*	Forward	AGAGGGAAATCGTGCGTGAC	[44]
	Reverse	CAATAGTGATGACCTGGCCG	
*rrsG*	Forward	GTTACCCGCAGAAGAAGCAC	[43]
	Reverse	CACATCCGACTTGACAGACC	
*lon-outer*	Forward	CAGGAGTTCTTACAGGTAGA	This study
	Reverse	CCACACTCCGCTGTAGGTGA	
*cpxR-outer*	Forward	CATCATCTGCGGGTTGCAGC	[13]
	Reverse	GATAATTTACCGTTAACGAC	
*sifA-*pKD3	Forward	GATTTAATCAATTATGTAGTCATTTTTACTCCAGTATAAGTGAGATTAATGTGTAGGCTGGAGCTGCTTC	This study
	Reverse	AGTACGTGAGTAAACCCTGAACGTGACGTCTGAGAAAGCGTCGTCTGATTATGGGAATTAGCCATGGTCC	
*sifA-*inner	Forward	ATGCCGATTACTATAGGGAATGGT	This study
	Reverse	CAACATAAACAGCCGCTTTGT	
*sifA-outer*	Forward	CATCCGCGGTAGTCCTTCTT	This study
	Reverse	AACAAATTGCCAGACGAGCG	
*asd*-pKD3	Forward	TGAAGGATGCGCCACAGGATACTGGCGCGCATACACAGCACATCTCTTTGGTGTAGGCTGGAGCTGCTTC	This study
	Reverse	TATCCGGCCTACAGAACCACACGCAGGCCCGATAAGCGCTGCAATAGCCAATGGGAATTAGCCATGGTCC	
*asd*-inner	Forward	CATGGTAGAGGAGCGCGATT	This study
	Reverse	TACCGCCCACAAAGGTCTTC	
*asd*-outer	Forward	GCGACGGAAATGATTCCCTT	This study
	Reverse	AAGCTACCCTTAAAGAATAGCC	
*pagL*-pKD3	Forward	AATTTTAAATATGTTAGCCGGTTAAAAATAACTATTGACATTGAAATGGTATGCTCAAACAGACATTACA	This study
	Reverse	CGGTGATTAATTACTCCTTCAGCCAGCAACTCGCTAATTGTTATTCAACTATGGGAATTAGCCATGGTCC	
*palL*-inner	Forward	CAGATCTCTTTTGCTGCGGG	This study
	Reverse	AAAAGCCCCAAAGTTCCAGC	
*pagL-outer*	Forward	TGGATGTGCCTGAACAACACT	This study
	Reverse	TTAGCCTCCCTGTCGCCATA	
*rfaL-*pKD3	Forward	TTTGGAAAGATTCATTAAAGAGACTCTGTCTCATCCCAAACCTATTGTGGGTGTAGGCTGGAGCTGCTTC	This study
	Reverse	CCTGATGATGGAAAACGCGCTGATACCGTAATAAGTATCAGCGCGTTTTTATGGGAATTAGCCATGGTCC	
*rfaL-inner*	Forward	ACAAGTTTAGGACTTCGCTGCC	This study
	Reverse	CAGAATGGTATTATGCGGACCG	
*rfaL-outer*	Forward	GCAGCGTTTCGAGGAACAAA	[27]
	Reverse	TCGTATCGGTTGATACCGGC	
*lpxE*	Forward	GAAAACTATATTAAGCATTCT	This study
	Reverse	GCCGTATTCCAGGAAGAACTTTGG

### Animals and ethics statement

All experiments conducted using mouse models were approved by the Jeonbuk National University (CBNU-2021-037) animal ethics committee following the guidelines of the Korean Council on Animal Care and Korean Animal Protection Law, 2007: Article 13. Six-week-old specific pathogen-free female BALB/c mice procured from Samtako, Osan, Korea, were maintained on a standard feeding regimen with antibiotic-free food and water ad libitum at the Animal Housing Facility of the College of Veterinary Medicine, Jeonbuk National University.

### In vitro study

In vitro infection of *Salmonella* JOL2782 and JOL2837 was conducted in RAW 264.7 cells, HEK293T cells, mouse intestinal epithelial cells (IEC-6), and Caco-2 cells. The attenuated *Salmonella* strains were grown overnight at 37 °C and recultured to log phage for 3 h (OD_600_: 0.4–0.6). The culture was centrifuged and prepared in phosphate-buffered saline (PBS). Cells grown to 60–70% confluence were infected with the wild-type (WT) *Salmonella* JOL401 strain and the attenuated strains at 40 multiplicity of infection (MOI). Inoculated cells without infection were used as a control. An accepted protocol for infection was performed in which monolayers were washed with PBS and treated with gentamycin (100 µg/mL) for 2 h to eliminate extracellular bacteria [15].

### Adhesion, invasion, and intracellular survival

The ability of the designed *Salmonella* vaccine delivery vehicles, viz. JOL2782 and JOL2837, to adhere and invade cultured mammalian cells such as RAW 264.7, HEK293T, IEC-6, and Caco-2 was assayed by adhesion and invasion assays. Bacteria were allowed to interact with cells for 30 min and washed with PBS three times to remove nonadherent cells. The adhered bacteria were counted by plating on Brilliant Green Agar (BGA) (BD Difco™) after treating cells with 0.25% Triton X-100. In the invasion assay, bacteria were incubated with cells for 2.5 h to invade the cells. The noninfected bacteria were eliminated by gentamycin treatment, and internalized bacteria were counted by plating on BGA after treating cells with 0.25% Triton X-100. For intracellular survival, cells were allowed to invade by bacteria for 2.5 h, washed, treated with gentamicin, and further incubated for 12 h. After incubation, monolayers were lysed with 0.25% Triton X-100 to release intracellular bacteria, serially diluted, and plated on BGA for counting.

### Stress survival assay

The intactness of our delivery vector was evaluated for the stress of pH and oxidative pressure. Survival was determined for the strains grown to log phage after overnight cultures. Strains were treated with acidic stress at pH 4.0 and with oxidative stress at 1 mM and 5 mM H_2_O_2_ for 30 min. The rate of survival based on the initial inoculum was assessed by plating on BGA [15] and confirmed the retention of a heterologous gene by heterologous antigen construct-specific primers.

### Expression of virulence genes under stress

The expression of virulence genes under the stress conditions of oxidative pressure and pH for 30 min was examined at the mRNA level. Total RNA was isolated and converted into cDNA (Elpis Biotech, Daejeon, South Korea) for quantification of virulence gene expression using qRT‒PCR. Thermal cycling conditions were set as preincubation at 95 °C for 5 min followed by 40 cycles of 30 s each at 95 °C, 56 °C, and 72 °C. The specificity of the PCR amplification and absence of contamination was confirmed by melting peak analysis with a temperature gradient of 0.1 °C s-1 from 65 °C to 95 °C. Gene expression was normalized against the housekeeping gene rrsG, and fold changes in expression levels are presented using the 2^−ΔΔCT^ method.

### Preparation of recombinant antigen protein and production of primary antibodies

For the production of the coating antigen, the consensus sequence from Influenza A H1N1 coding the HA globular head region was cloned into the pET28 ( +) expression vector (Novagen, San Diego, CA, USA) and transformed into the *Escherichia coli* BL21 (DE3) strain (Novagen) for protein expression. The expressed protein was purified by a Ni-NTA chromatographic column (Bio-Rad, California, USA), dialyzed against PBS, quantified by a Bradford assay [21], and stored at −80 °C until use. The recombinant protein was injected into the rabbit along with Freud’s complete adjuvant and bolstered with Freud’s incomplete adjuvant at 2-week intervals, and the hyperimmune sera were collected for Western blotting and IFA. Similarly, the crude soluble protein was extracted from *Salmonella* WT culture, immunized the rabbit, and collected the hyperimmune sera for anti-*Salmonella* rabbit antibodies. In addition, the recombinant protein was used to coat the ELISA plate for the IgG assay.

### Western blotting

The expression of the heterologous antigen in RAW 264.7 cells was conducted in 6-well plates by infection with JOL2782 and JOL2837 at 40 MOI for 2 h. Noninvading *Salmonella* was irradiated by gentamycin treatment for 2 h. After 48 h of incubation, the cells were harvested using 150 μL of RIPA lysis buffer. Cells were subjected to 2–4 cycles of sonication for 5 s with a 60 s gap at 50% amplitude. The supernatant was applied to a 12% gel and transferred to a PVDF membrane (Immobilon^R^-P; Millipore, Ireland). Proteins in the membrane were allowed to interact with primary antibodies from rabbits at a 1:500 dilution (polyclonal antibodies raised against the recombinant HA protein expressed in *E. coli*) and with anti-rabbit IgG HRP antibody (Southern Biotech, Birmingham, AL, USA) at 1:6000. The chemiluminescent substrate was added, and images were obtained and documented (Cytiva, Marlborough, MA, USA).

### Immunofluorescence assay (IFA)

Protein expression was confirmed by IFA in RAW 264.7 cells by transfection with JOL2782 and JOL2837 at 40 MOI for 2 h. Noninvasive *Salmonella* was eradicated by gentamycin treatment, and the media was replaced with DMEM containing 2% FBS. Cells were incubated for 48 h to allow protein expression. Then, the cells were fixed with chilled 80% acetone at −20 °C for 10 min. The cells were permeabilized using 0.1% Triton X-100, blocked with 3% BSA, and incubated with primary antibodies raised against recombinant HA protein in rabbits at a 1:200 dilution at 4 °C overnight. After washing, the cells were stained with Alexa Fluor 488-conjugated donkey anti-rabbit IgG (Invitrogen, Waltham, MA, USA) at 1:5000. DAPI was used to stain the nucleus. Cells were observed under a Leica fluorescence microscope (Leica Biosystems, Wetzlar, Germany).

### In vivo experiment

A safety assessment test was performed in 6-week-old female mice by administering *Salmonella* JOL2782 and JOL2837 at a respective concentration of 1 × 10^7^ CFU and 1 × 10^8^ CFU per mouse prepared in 100 μL of PBS for intramuscular (IM) inoculation and peroral administration. Ten mice were allocated to each group. An equal number of mice received WT JOL401 for the survival assay, and the control group was injected with PBS. Two mice per group were sacrificed at 1, 3, 7, 14, and 21 days post-inoculation (dpi).

### General observation

Mice inoculated with the delivery vectors were observed daily for adverse effects such as disease symptoms, mortality, and morbidity. Local reactions and adverse effects, such as abnormalities, deterioration in general health, and reduction of feed intake, were monitored. Body weight was measured once a day in the morning. Along with these, evidence of diarrhoea, ruffled (ungroomed) fur, or irritability was recorded.

### Survival assay

For the survival assay, mice were injected with *Salmonella* JOL2782, JOL2837, WT JOL401, or PBS as a placebo and monitored daily for mortality and morbidity up to 21 days post-inoculation.

### Localization of *Salmonella* in vital organs

For bacterial load assessment in vital organs, mice were euthanized by cervical dislocation, and organs were collected aseptically. Half of each collected organ was homogenized separately in PBS using a mechanical homogenizer (IKA T 10 basic ULTRA-TURRAX, Germany) and plated on BGA at tenfold serial dilution. Representative colonies were confirmed for ST strains by PCR using ST-specific primers for outer membrane protein-coding genes of JOL401 and further verified for respective strains by using deletion-specific primers. Further confirmation was performed by immunohistochemical assays as described later in the methods.

### Immunohistochemical assay

Immunohistochemical staining was performed to detect the localization of the delivery vector using anti-*Salmonella* rabbit antibodies. For this experiment, the remaining halves of the organs were harvested in 10% normal formaldehyde solution and preserved before processing. Briefly, tissues were embedded in paraffin and sectioned. Specimens were deparaffinized using xylene and hydrated by grading in ethanol and distilled water. Antigen retrieval was achieved by heating at 100 °C for 30 min in citrate buffer (pH 6), and inhibition of endogenous peroxidase activity was performed by adding 0.3% methanolic H_2_O_2_. Specimens were labelled with primary antibodies raised against the crude soluble protein of *Salmonella* in the rabbit at a 1:200 dilution and treated with secondary goat anti-rabbit IgG HRP antibody (1:250; Southern Biotech, Birmingham, AL, USA). Finally, colour development was achieved using 3,3' diaminobenzidine (DAB) substrate (Sigma-Aldrich, Steinheim am Albuch, Germany) [22].

### Bacterial dissemination in the environment

All immunized mice were monitored for biological containment of *Salmonella.* Bacterial dissemination from the inoculum was investigated by the culture method and confirmed by PCR. For this, faecal samples were collected at 1, 3, 7, 14, and 21 dpi, suspended in buffered peptone water (BD Difco™) and pre-enriched for 18 h at 37 °C. Then, the samples were transferred to Rappaport-Vassiliadis R10 broth (BD Difco™), an enrichment medium, and incubated for 24 h at 42 °C. Bacterial quantification was accomplished by streaking the samples on BGA at different dilutions and counting the colonies. Colonies were further confirmed by PCR using ST-specific primers and further verified for the respective strains by using deletion-specific primers.

### Tissue damage by *Salmonella*

Comparative tissue damage caused by the vaccine delivery vector was monitored in the liver, spleen, lung, and brain by a histopathological study using haematoxylin and eosin staining. Two mice per group were sacrificed on the third day, and the spleen, liver, lung, and brain were harvested to confirm the localization and extension of damage caused by *Salmonella* in the respective organs. Tissues were fixed and processed for H&E staining based on the standard procedure explained elsewhere. The morphological dimension of tissues for inflammatory signs, such as macrophage infiltration, accumulation, distortion in the liver, and distorted red pulp areas in the spleen, was noted.

### Enzyme-linked immunosorbent assay (ELISA)

Total IgG was measured from sera collected at 14 and 21 dpi by ELISAs. Briefly, 96-well high-binding polystyrene plates (Greiner Bio-One, Kremsmünster, Austria) were coated with recombinant proteins at 5 μg/mL in carbonate-bicarbonate buffer, pH 9.6, and incubated at 4 °C overnight. The wells were blocked with 200 μL of 5% skim milk at 37 °C for 1 h and washed with 0.1% PBST. Serum samples were added, incubated at 37 °C for 1 h, and washed as above. HRP-conjugated goat anti-mouse IgG antibody at 1:5000 (Southern Biotech, USA) was added and incubated at 37 °C for 1 h. Finally, colour was developed with 100 μL of freshly prepared OPD substrate in phosphate-citrate buffer (pH 5.0) containing H_2_O_2_ at room temperature for 5–10 min in the dark. The reaction was stopped by adding 50 μL of 3 M H_2_SO_4_, and the optical density (OD) was measured at 492 nm in a microplate reader (Tecan, Zürich, Switzerland).

### Cytokine measurement

The endotoxicity of the delivery system was measured through the levels of inflammatory cytokines such as TNF-α, IFN-γ, and IL-1β. Spleens were harvested at 3 dpi, and RNA was extracted using RiboEX (GeneAll, Seoul, South Korea). Then, cDNA was synthesized from extracted RNA (Elpis Biotech, Daejeon, South Korea). Cytokine levels were measured using the primers listed in Table 2. The specificity of the PCR amplification and absence of contamination was confirmed by melting peak analysis. Changes in mRNA levels were determined by the 2^−ΔΔCT^ method using β-actin as an internal control.

### Statistical analysis

Data analysis was performed by Student’s *t* test and ANOVA using GraphPad Prism 9.0 software (GraphPad, San Diego, CA, USA). A *p* value < 0.05 was considered significant. Data in graphs are presented as the mean ± SD with *p < 0.05; ** *p* < 0.01; *** *p* < 0.001; and **** *p* < 0.0001. The statistical test employed and the number of animals are indicated in the figure legends.

## Results

### Construction of the *Salmonella* delivery strains

The construction of *Salmonella* delivery strains was accomplished by the λ red recombination method. The attenuated *Salmonella* strains with deletions of *lon* and *cpxR* were engineered to construct the delivery system JOL2782 with *sifA* deletion to confer defective SCV into the host system. Similarly, JOL2837 was further attenuated by the deletion of *rfaL* and substitution of *lpxE* for *pagL* for depleted O-antigen ligase and altered lipid A (Figure 1). Both strains were auxotrophic mutants and required *asd* gene complementation to replicate, which was complemented by the *asd* + plasmid vector. The engineered constructs were confirmed using the respective flanking primers (Additional file 1).

### Adhesion, invasion, and intracellular survival

**Figure 2:**
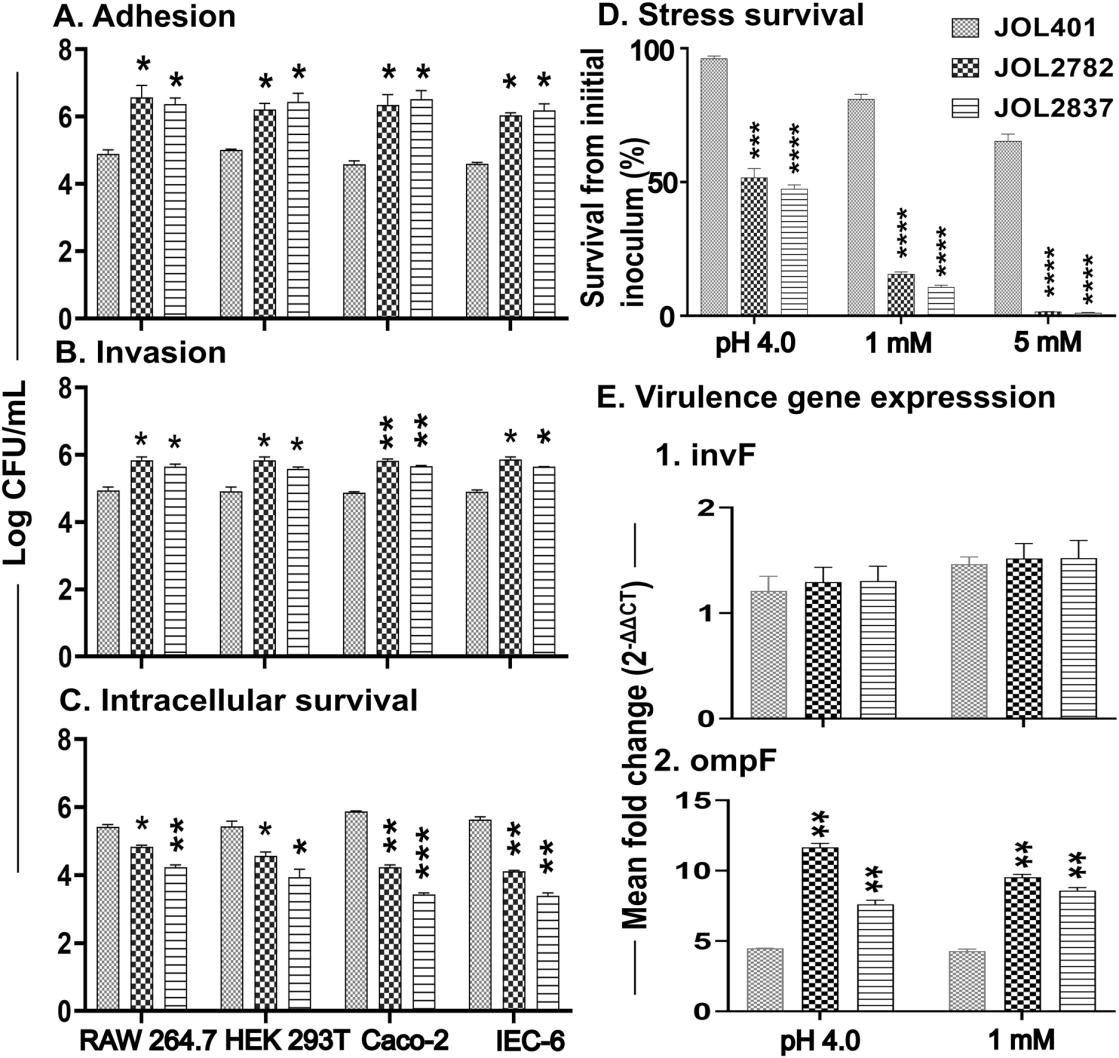
**In vitro assessment of adhesion, invasion, intracellular survival, and stress**. **A**. Adhesion, **B**. Invasion, and **C**. Intracellular survival. The strengths of adhesion, invasion, and intracellular survival of JOL2782 and JOL2837 were compared against those of the *Salmonella* JOL401 WT strain using RAW 264.7 cells, HEK293T cells, Caco-2 cells, and IEC-6 cells. Monolayer cells were infected with each strain at 40 MOI. The adhesion and invasion of each strain were assessed after 30 min and 2.5 h of incubation. Intracellular survival was evaluated 12 h post-infection. **D**. Stress survival of *Salmonella* WT strain JOL401 and attenuated strains JOL2782 and JOL2837 exposed to acid and oxidative H_2_O_2_ stresses. The susceptibility of strains was compared after 30 min of exposure. The survival of strains was determined based on the respective initial inoculum. E. Expression of virulence genes. *Salmonella* strains JOL401, JOL2782, and JOL2837 were exposed to the stress of 1 mM and 5 mM H_2_O_2_ at pH 4.0 for 30 min, and RNA was extracted for real-time PCR analysis. The expression of selected virulence genes was evaluated by normalizing against the housekeeping rrsG gene. Data were analysed by multiple unpaired t tests and are presented as *p < 0.05, **p < 0.01, ***p < 0.001, and ****p < 0.0001.

In vitro susceptibility to attenuated *Salmonella* in comparison with the wild-type strain was studied in mouse and human cell lines, such as RAW 264.7, HEK239T, Caco-2, and IEC-6 cells. Significantly higher levels of adhesion and invasion were noted for both attenuated strains in comparison with the WT strain in all tested cell lines (Figures 2A, B). This increased adhesion and invasion ensure successful internalization of the delivery strains and release of the plasmid that carries immunogens. Intracellular survival is directly proportional to vaccine deliverability, which incurs toxicity when above a threshold level. Both attenuated strains exhibited optimal intracellular survival, attributed to appropriate delivery of the foreign antigen. Comparatively, JOL2782 showed higher survival than JOL2837 (Figure 2C), which is anticipated due to a higher degree of deliverability.

### Stress survival

Vaccine delivery strains undergo various stresses in the host system. In comparison with the WT strain, the attenuated delivery strains displayed a significant reduction in survival rate from acidic and oxidative stress (Figure 2D). Oxidative stress induced by H_2_O_2_ at a 1 mM concentration drastically reduced viability but was not lethal for the attenuated strains. In parallel, the viability was decreased by nearly 50% at pH 4.0. The result indicates the moderate and safe viability of delivery strains in intracellular oxidative stress and under gut acidity conditions.

### Virulence gene expression

The expression of virulence genes related to adhesion and invasion was determined under stress conditions of pH 4.0 and H_2_O_2_ oxidative stress. The expression of *invF* remained equal to that of the wild-type; however, *ompF* expression was significantly enhanced in both delivery strains. *ompF* was more highly upregulated in JOL2782 than in JOL2837 (Figure 2E). It has been hypothesized that upregulation of *ompF* perhaps contributed to the improved adhesion of these strains.

### In vitro expression of heterologous antigen

**Figure 3:**
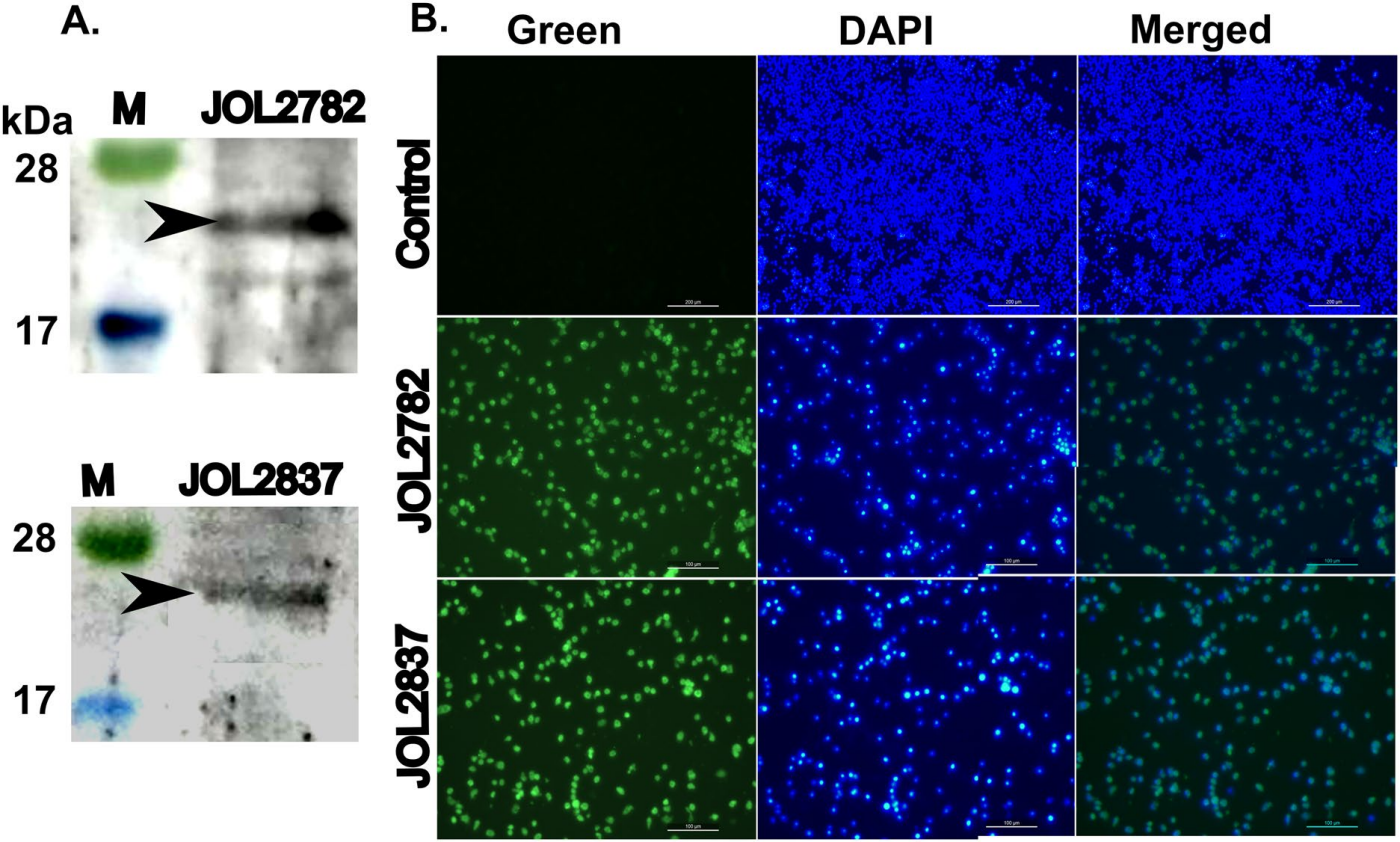
**Characterization of protein expression in RAW 264.7 cells**. Murine macrophage RAW 264.7 cells were infected with the vaccine strains JOL2782 and JOL2837 or the control. Protein expression was determined by A. Western blots and IFA. **A**. A Western blot image demonstrates the band representing the expression of the recombinant protein (22 kDa) in RAW264.7 cells. **B**. IFA was performed using rabbit antisera against the recombinant protein. Bright green fluorescence in the JOL2782- and JOL2837-infected cells indicates protein expression. Blue represents the nucleus stained by DAPI. No fluorescence was detected in the control.

The expression of the recombinant protein by the payload delivery system was validated in RAW 264.7 cells by Western blotting and IFA. Rabbit antisera interaction in Western blotting confirmed the protein expression in murine macrophages (Figure 3A). In IFA, green indicates foreign antigen expression, and blue (DAPI) indicates viable macrophages. The merged image depicting the emission of green fluorescence in the transfected cells with the attenuated strains establishes the expression of foreign antigen genes (Figure 3B). It depicts the suitability of the attenuated strains as a delivery system.

### General observation and survival

**Figure 4:**
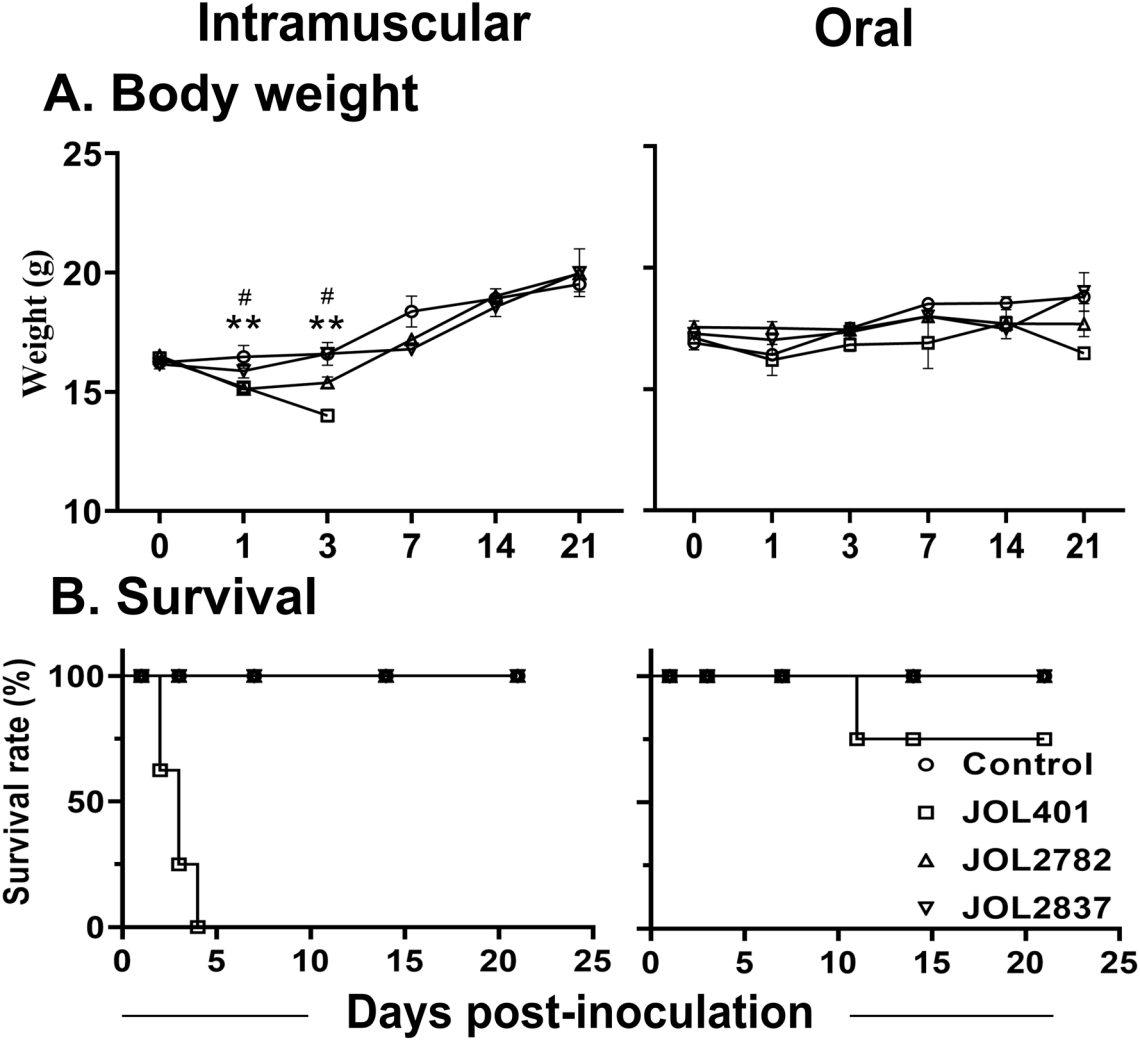
**Postimmunization evaluation of mice**. Mice were administered JOL401, JOL2782, JOL2837, or a PBS control via intramuscular inoculation and the peroral route. **A**. Body weight changes were noted up to 21 days post-inoculation. **B**. Survival rate curve for mice. The survival of mice was observed for up to 21 days. A bacterial concentration of 1 × 10^7^ CFU/mouse in PBS was inoculated into left thigh muscles for the IM route, and 1 × 10^8^ CFU/mouse was administered through the mouth for the PO route. All JOL401 IM-inoculated mice died within 4 dpi. The level of significance was *p* < 0.05 as measured by multiple unpaired t tests. The signs * and # denote a significant difference in comparison with the control group at the respective time point for the JOL401 and JOL2782 strains, respectively.

Mice inoculated with the attenuated strains showed the absence of local lesions, ulceration, abscess, or necrosis at the injection site, and no adverse effects or reduction in feed intake was observed. The body weight change in the mice inoculated with the attenuated strains was comparable to that in the naive mice in both routes of infection (Figure 4A). Moreover, evidence of diarrhoea, ruffled (ungroomed) fur, or irritability was not found in the mice inoculated with the attenuated strains. However, the mice inoculated with the wild-type JOL401 strain exhibited prominent ruffled fur, irritability, lethargy, hunched posture, and grave illness.

Neither intramuscular nor peroral administration of the attenuated delivery strains caused any disease symptoms or mortality in the mice during the entire experimental period. All mice inoculated with JOL401 by IM exhibited peracute infection and succumbed to mortality at Day 4 post-inoculation (Figure 4B). With the PO route, mortality was not observed until 11 dpi for the WT strain, but considerable disease symptoms were observed. The in vivo mortality and morbidity assessment distinctly revealed the nonpathogenicity of the present delivery strains even at a tenfold higher dose of inoculation.

### Bacterial colonization and persistence in internal organs

**Figure 5:**
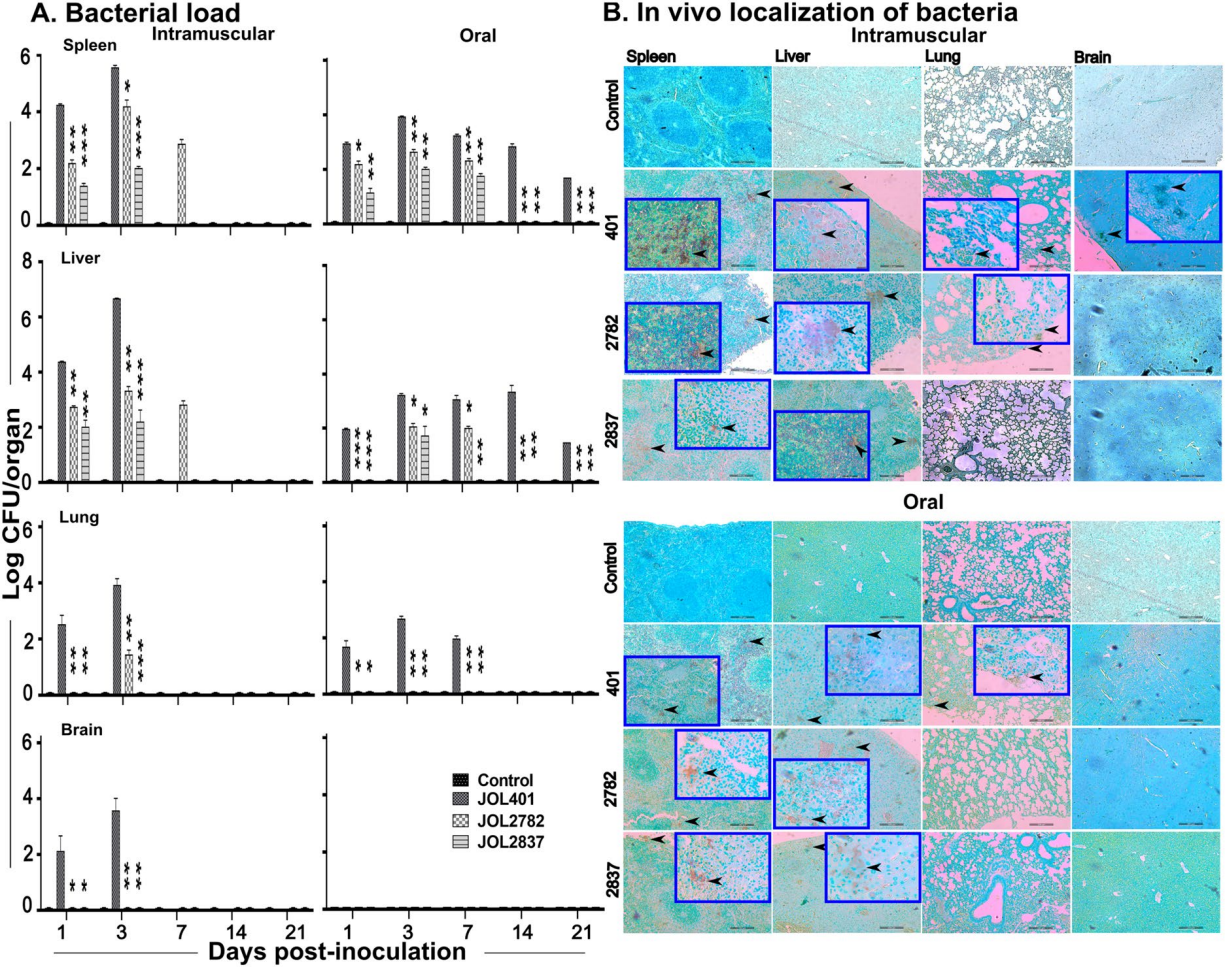
**Evaluation and confinement of bacterial load in different internal organs post-inoculation.** Mice were administered *Salmonella* by the IM and PO routes. **A**. Bacterial load. The spleen, liver, lung, and brain were aseptically collected. Samples were homogenized in PBS, and the bacterial load was quantified in *Salmonella* selective media, BGA. On 4 dpi, all JOL401 IM-inoculated mice died, and the bacterial load was not analysed. For both JOL2782 and JOL2837, bacteria were not recovered from the tested organs at 14 and 21 dpi. Data were analysed by multiple unpaired t tests and are presented as *p < 0.05, **p < 0.01, and ***p < 0.001. **B**. Immunohistochemical staining of tissues (IHC). Distribution and delivery of *Salmonella* in mouse organs were evaluated by IHC staining of tissue sections at 3 dpi. Brown spot regions indicated by black arrowheads represent the bacteria inhabiting the tissues. Images were obtained at 100X magnification, and inset images were obtained at 400X magnification. The scale bar indicates 200 μm.

Microbial loads in internal organs such as the spleen, liver, lung, and brain from the placebo control group were negative for *Salmonella* recovery examined for both the IM and PO routes. Through the IM route, the spleen and liver were populated by JOL401, JOL2782, and JOL2837 from 1 dpi. With the IM route, JOL2782 lasted for 7 dpi in the spleen and liver, while JOL2837 was cleared from both organs at this time point (Figure 5A). Conversely, the IM-inoculated WT strain was dispersed in all tested organs. In the case of PO inoculation, only the WT strain was recovered after 1 dpi from the spleen, liver, and lung and remained until 21 dpi in both the spleen and liver. JOL2782 was noted in the spleen and liver at 7 dpi, while JOL2837 was cleared from the spleen at this time point. This finding reveals that attenuated *Salmonella* effectively reached the lymphoid organs and thereby increased the probability of antigen delivery. The WT strain administered by the IM route invaded the brain by crossing the blood‒brain barrier (Figure 5A), but the test strains were unable to do so. Overall, the results indicate reduced infectivity in both attenuated strains, but they retained desirable infectivity to deliver the foreign antigen to the immune cells in secondary lymphoid organs. Comparatively, JOL2782 is more feasible and viable in various organs than JOL2837, indicating better deliverability.

Immunohistochemical staining of the internal organs developed brown spots post-DAB staining. It is a confirmatory signal of the presence of inoculated *Salmonella,* where it is encased by the antigen-presenting cells (Figure 5B). Localization of the JOL401 WT strain was detrimentally prominent, while attenuated strains showed moderate and diffusive distribution. This result further substantiates the CFU assessment results that these delivery strains exhibit nondeleterious reach and viability in vital organs.

### Bacterial confinement

Faecal samples monitored at regular intervals represent bacterial dissemination in the environment. Discharge of WT JOL401 was noted for a prolonged period, while both attenuated strains were confined to the host, without any residual discharge with IM inoculation. With the oral route, the WT strain was found up to 14 dpi, whereas both delivery strains were noted at a trivial number only up to 3 dpi (Table 3). To date, the attenuated strains have been confined within the body and do not disseminate to the environment via faeces.

**Table 3:** **Bacterial shedding in the faecal samples**. Bacterial load is represented as the average bacterial dissemination in faecal samples (CFU/mg).

**Group**	**Day 1**	**Day 3**	**Day 7**	**Day 14**	**Day 21**
Intramuscular					
Control	0	0	0	0	0
JOL401	3.6 × 10^5^	8 × 10^5^	Died		
JOL2782	0	0	0	0	0
JOL2837	0	0	0	0	0
Oral					
Control	0	0	0	0	0
JOL401	0	1.2 × 10^3^	3.3 × 10^5^	8.9 × 10^3^	0
JOL2782	0	25	0	0	0
JOL2837	0	15	0	0	0

### Morphological changes in the spleen and liver

**Figure 6:**
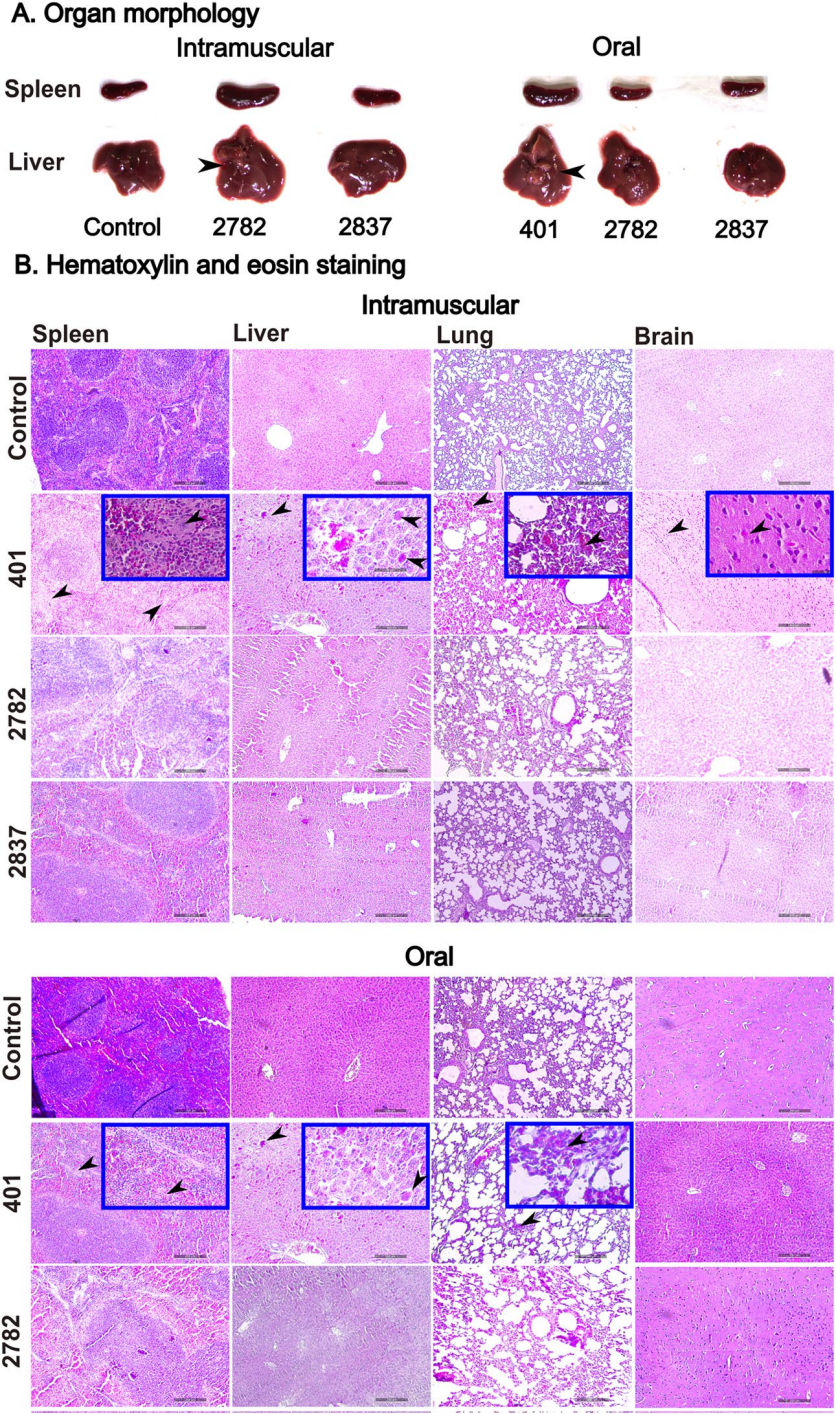
**Bacterial invasion in internal organs.** **A**. Morphological changes in the spleen and liver. Mice were sacrificed at 14 dpi, and the spleen and liver were harvested. Remarkable splenomegaly was observed in mice inoculated with JOL401 via the oral route and with JOL2782 by the IM route. Hepatomegaly was observed with whitish lesions denoted by black arrowheads that were prominent in mice subjected to oral injection of JOL401, and a small lesion was noted in mice intramuscularly inoculated with JOL2782. **B**. Histopathological analysis. Mice were inoculated with *Salmonella* strains, and the effect on the organs was investigated and compared with the organ samples of mice receiving PBS as a placebo. Histopathological changes in the spleen, liver, lung, and brain collected at 3 dpi were studied. Prominent tissue dispersion of red pulp in the spleen as an indication of inflammation was observed in mice inoculated with JOL401 in comparison with attenuated strains and is represented by black arrowheads. Mild to moderate infiltration of inflammatory cells was observed in the liver, denoted by black arrowheads. A similar accumulation of macrophages as an indication of inflammation was noted in the lung and brains of JOL401 WT strain-inoculated mice. Images were obtained at 100 × magnification, and inset images were obtained at 400 × magnification. The scale bar indicates 200 μm.

Morphological changes in secondary lymphoid organs, such as the spleen and liver, were recorded. The organs of naive mice were processed as a reference control, and there was no alteration in the normal physiological state. In the case of IM inoculation, there was mild or no substantial splenomegaly and hepatomegaly observed in JOL2782 and JOL2837, respectively, at 14 dpi, whereas the JOL401 mice did not survive after 4 dpi. After oral administration, inflammatory lesions and organ enlargement were observed only with JOL401, but neither attenuated strain-induced adverse effects on the organs (Figure 6A). Organ morphological assessment revealed that both tested strains were safe compared with the WT strain. JOL2837 is safer than JOL2782, as it exhibited negligible impairment and improved safety due to its lower endotoxicity and reduced intracellular survival.

### Histological analysis

Histopathological assessment of tissue sections of the spleen, liver, lung, and brain collected at 3 dpi was performed to determine abnormalities compared with those of the naive mice receiving PBS. In the mice subjected to IM inoculation with JOL401, prominent tissue damage was ascertained, whereas with both attenuated strains, negligible damage was observed (Figure 6B). Similarly, after oral administration, the WT strain exhibited considerable inflammatory tissue alteration, while the mice that acquired the tested strains showed healthy tissues. This histopathological analysis supports the previous results, and both strains fulfil the eligibility criteria of safety as delivery strains.

### Humoural response

**Figure 7:**
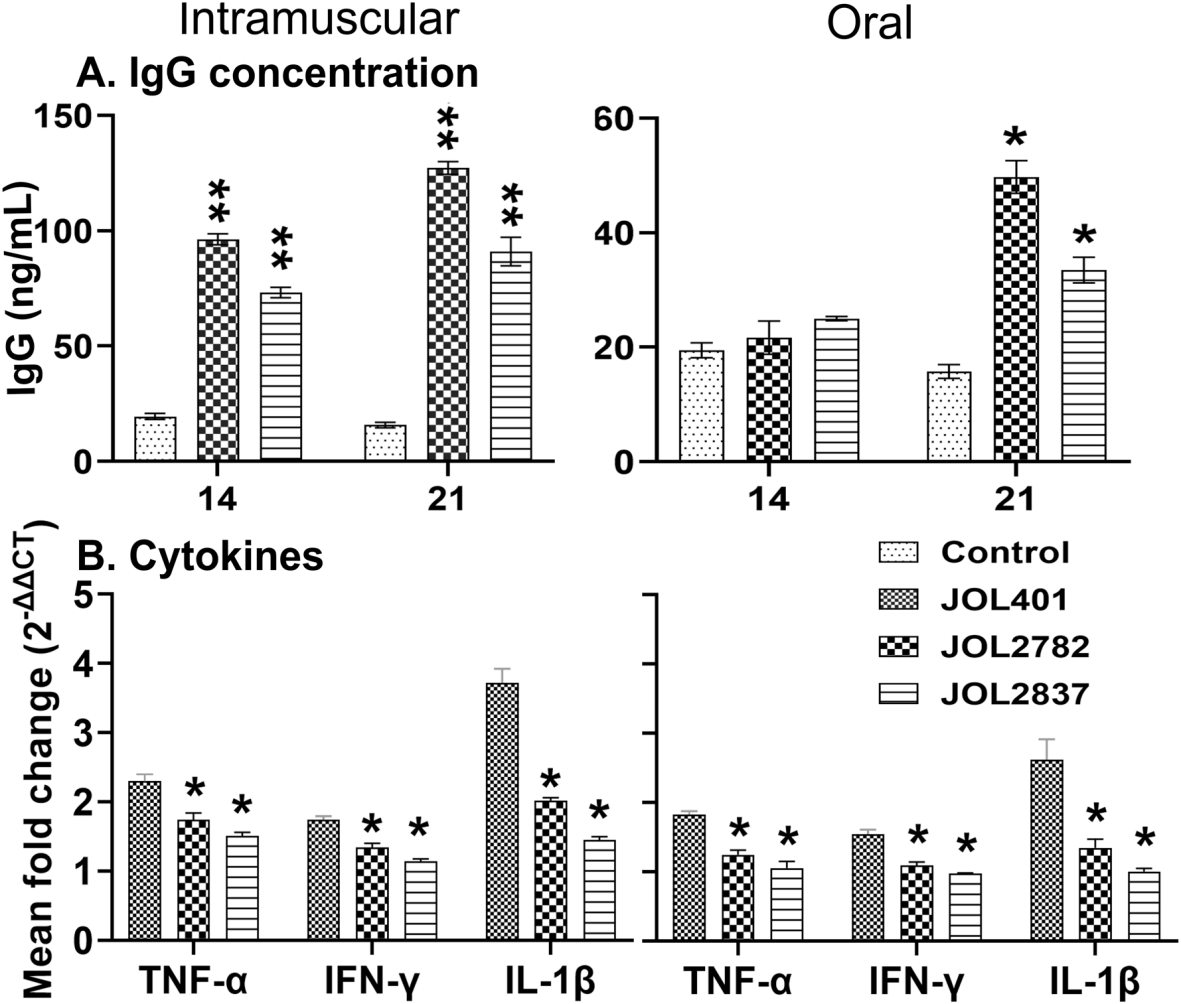
**Expression of humoral and cytokine responses.** **A**. Total IgG antibody response. Mouse serum was collected, and total IgG antibodies against the recombinant protein were assessed at 14 and 21 days post-inoculation. The level of IgG was compared with that of the PBS control group. **B**. Expression of cytokine genes. The spleen was harvested at 3 days post-inoculation, and mRNA was extracted to determine the expression levels of the inflammatory cytokines TNF-α, IFN-γ, and IL-1β by quantitative real-time PCR. A significant difference against the WT strain for the expression of genes was measured and analysed by multiple unpaired t tests and is presented as *p* > 0.05, *p < 0.05, **p < 0.01, and ***p < 0.001.

The antigen-specific antibody response in the mouse sera was determined by ELISAs using recombinant proteins. Sera collected at 14 and 21 dpi were analysed for IgG antibodies. The JOL2782- and JOL2837-immunized mice mounted an antigen-specific antibody response to recombinant protein, and the increase in IgG was significant from 14 dpi for the IM route of inoculation. Oral immunization induced a late and minimal response, highlighting the requirement for a booster dose (Figure 7A). Through IgG measurement, JOL2782 showed higher induction of humoural response than JOL2837, as it possesses higher intracellular survival.

### Endotoxicity assessment

The inflammatory cytokines TNF-α, IFN-γ, and IL-1β were evaluated to measure the endotoxicity of the attenuated strains. Compared with that of the WT strain, the level of endotoxicity was significantly lower for the attenuated strains, which was reflected by the significant fold decreases in TNF-α, IFN-γ, and IL-1β for IM inoculation (Figure 7B). There was a considerable reduction in cytokine expression in the mice inoculated through the oral route. JOL2837 induced lower cytokine expression than JOL2782, which reflects the minimum endotoxicity of this strain. The determination of cytokines indicates moderate induction of the innate immune response with significantly reduced inflammation by the delivery strains.

## Discussion

Vaccines for zoonotic illnesses in food animals, domestic pets, and even wildlife have significantly reduced the prevalence of zoonotic diseases in humans. *S. enterica* causes a spectrum of diseases in animals and humans, ranging from mild infection to fatal infection. For a cost-effective, highly efficacious vaccine delivery system, *Salmonella* is an ideal lead due to its mode of pathogenesis and invasiveness to multiple sites and organs in the host. However, different strains of *S. enterica* serovar Typhimurium cause enteric infections in humans and other warm-blooded animals, limiting their use in the animal industry as well as in human application. Live attenuated *Salmonella* is an attractive agent to be developed as a vaccine or drug delivery system for payload immunogens and heterologous antigens. Genetically engineered ST as a vaccine for poultry and swine is gaining popularity, but bacterial shedding is the limiting factor for successful usage. In our previous studies, *lon* and *cpxR* mutant *Salmonella* exhibited increased bacterial polysaccharide production [13, 23, 24] and increased macrophage‒bacteria uptake [25]. This characteristic was anticipated to generate a better payload of the desired antigen in the target region for enhanced response. Based on this, we designed attenuated strains by the removal of *lon* and *cpxR* to achieve efficient adhesion and invasion without systemic infection [13, 26, 27]. In addition to deletion of *lon* and *cpxR*, the present study investigated the safety of the attenuated *Salmonella* strains JOL2782 (*Δlon*, *ΔcpxR*, *ΔsifA*, and *Δasd*) and JOL2837 (*Δlon*, *ΔcpxR*, *ΔrfaL*, *ΔpagL::lpxE*, and *Δasd*) developed by deletion of multiple genes corresponding to virulence, internal integrity in host cells, and LPS structure (Figure 1).

The removal of *sifA* disrupts the integrity of the SCV, and this mutation has been reported to cause defective intracellular replication [14, 28, 29]. Considering the critical role of *sifA* in *Salmonella* virulence and presentation in host cells, the *Salmonella* strain JOL2782 was developed. In parallel, *Salmonella* JOL2837 was designed as a delivery vector by replacing *pagL* with *lpxE* for retarded endotoxicity, and deletion of the *rfaL* gene corresponded to the O-antigen. Deletion of the *rfaL* gene has augmented sensitivity to complement-mediated cell death [30] and uptake by dendritic cells and reduced intracellular proliferation [31] yet maintained antigen presentation for sufficient elicitation of protective immune responses [32]. As the strain still contains intact outer and inner core LPS along with structurally changed lipid A, it possesses immunogenicity, humoural responses, and inflammatory responses [5].

The safety aspects of attenuated strains were studied based on virulence and intracellular survival with respect to the WT strain. Attenuation of *Salmonella* enhanced adhesion and invasion in murine and human cell lines in vitro (Figures 2A, B). In concordance, the attenuated strains exhibited reduced intracellular survival in comparison with the WT strain (Figure 2C). The *lon* deletion in both attenuated strains resulted in improved adhesion and invasion; moreover, the *cpxR* deletion was attributed to reduced intracellular survival. JOL2837 is more sensitive than JOL2782 to macrophage killing due to its additional deletion of endotoxicity-related genes. As a whole, improved invasion and reduced intracellular survival denote higher vaccine delivery potential. As vaccine delivery strains, they have vast dynamics and alterations in gene expression to sustain oxidative and acid stress in the host system. Such stresses minimize the survivability of the attenuated strains (Figure 2D), but a stressful acidic environment has exaggerated the expression of biofilm, stress, and virulence genes [33]. Modulation of virulence genes in attenuated strains (Figure 2E) indicates their improved adhesion and strengthens the delivery strain phenotype. The higher expression of virulence genes and low intracellular survival allow viability of the attenuated strains for a limited period in the host, ensuring the appropriate delivery of foreign antigens and timely discharge of these strains without any undesirable effect (Figure 2).

SPI-1, the determining factor for adhesion and invasion, is negatively regulated by the *lon* gene [26, 34–36]. As we observed from the enhanced adhesion and invasion of JOL2782 and JOL2837 in cell lines, the expression of genes related to adhesion and invasion, such as *invF* and *ompF*, was determined. The level of *invF* was the same as that of the WT strain, whereas the *ompF* level was significantly elevated (Figure 2E). The bacterial outer membrane protein *ompF* is a porin that is recognized by macrophages, leading to phagocytosis [37, 38]. This molecule is crucial to bacterial homeostasis, structural integrity, and virulence. Importantly, *ompF* plays a vital role in adhesion and invasion [37]. Thus, the result implies that the improved adhesion and invasion of these delivery strains were independent of *invF* but dependent on the upregulation of *ompF*.

As a candidate heterologous antigen, the H1N1 viral sequence was chosen, cloned into the pJOL204 expression vector, and transformed into our delivery system. The expression of the recombinant protein in murine macrophages was confirmed by Western blotting and IFA. Protein bands in Western blotting and bright green fluorescence in IFA verified the delivery of the target gene along with its expression in the host cells (Figures 3A, B). Mice inoculated with the WT strain by the IM route exhibited lethargy, ruffled fur, sickness, decreased food and water intake, and 100% mortality. In the case of inoculation with the attenuated strains, all the mice survived through the end of the experiment without any considerable morbidity (Figure 4). The results clearly show that these delivery candidates are safe at a higher dose than the lethal dose of WT, which is an indispensable characteristic of a delivery system. The absence of bacterial dissemination from the host to the environment was confirmed by the culture method (Table 3) and PCR detection from faecal samples of mice inoculated with the attenuated strains.

The attenuated *Salmonella* JOL2782 and JOL2837 strains were localized to the lymphatic system without crossing the blood‒brain barrier and induced a protective humoural response. Both attenuated strains were persistent in the spleen and liver for a short time, up to 7 dpi, without any adverse effects in the immunized mice, leaving sufficient time for intracellular delivery of plasmid cargo and concluding safe administration of the vaccine. Retention of strain JOL2837 for a shorter period in the host organs revealed that it is less immunogenic but safer than JOL2782 (Figure 5A).

*Salmonella* JOL401 inoculation via the IM and oral routes resulted in prominent inflammation and macrophage infiltration in the vital organs, as revealed by histopathological analysis, while the absence of such inflammatory signs in the mice inoculated with the attenuated strains demonstrated the safety of the delivery strains (Figure 6). Intramuscular inoculation of the WT strain was lethal and killed all the mice within 4 days, whereas there were mild or no distinguishable changes noted in secondary lymphoid organs for the JOL2782- and JOL2837-inoculated mice. Splenomegaly and hepatomegaly were prominent in the mice administered WT JOL401 via the oral route, but there were no remarkable changes in the mice inoculated with the attenuated strains, exhibiting the safety potential of the delivery system (Figure 6A). In IgG determination, both delivery strains ignited a humoural response against foreign antigens. Comparatively higher induction of IgG by JOL2782 at 14 and 21 dpi (Figure 7A) was observed than that of JOL2837. The result was consistent with the bacterial viability in organs, and higher IgG induction was achieved due to higher survival in the host system than that of JOL2837.

*Salmonella* infection triggers a signal for the expression of a diverse range of inflammatory cytokines, such as TNF-α, IL-1β, and IFN-γ. These mediators are essential for host resistance to *Salmonella* invasion, but excessive production can cause a severe adverse inflammatory response and even fatality as a consequence of endotoxic shock [39, 40]. Among the two strains, JOL2837 showed less stimulation of endotoxicity-related cytokines due to its defective lipid A, which corresponds to the deletion of *rfaL* and *pagL*::*lpxE*. The reduced endotoxicity led to less TLR4-based recognition and resulted in lower cytokine production (Figure 7B). This observation supports the higher safety of JOL2837 and JOL2782 as drug delivery systems.

In conclusion, we established two delivery systems of *Salmonella* strains that were substantially attenuated by the deletion of key virulence-associated genes, resulting in improved invasiveness and noncytotoxicity in the in vitro analyses. The systems also did not result in any conspicuous disease conditions or faecal discharge into the environment. We observed that these strains effectively reached the spleen, a major lymphatic organ, and the liver, both of which are prime sites for antigen presentation and immune activation. Additionally, they were rapidly cleared from internal organs, increasing their safety to the host. The expression of inflammatory cytokines was reduced to an optimum level to induce immunogenicity with significantly lower inflammation. Higher immunogenicity and lower cytotoxicity and endotoxicity are hallmarks of a promising delivery strain. These findings imply that the designed delivery strains are safe and efficacious immunization agents capable of eliciting an immune response. Further dose optimization is perhaps needed for different hosts to use these strains for delivery.

## Supplementary Information

**Additional file 1: **Confirmation of deletion of genes in *Salmonella* Typhimurium. The deletion of genes for the engineered Salmonella constructs was confirmed using the respective flanking primers.
