# Supplementary figures and images for: Safety assessment of compliant, highly invasive, lipid A-altered, O-antigen-defected Salmonella strains as prospective vaccine delivery systems
Source: Vet Res. 2022 Oct 1;53:76. doi: 10.1186/s13567-022-01096-z (PMC9526937; doi:10.1186/s13567-022-01096-z)

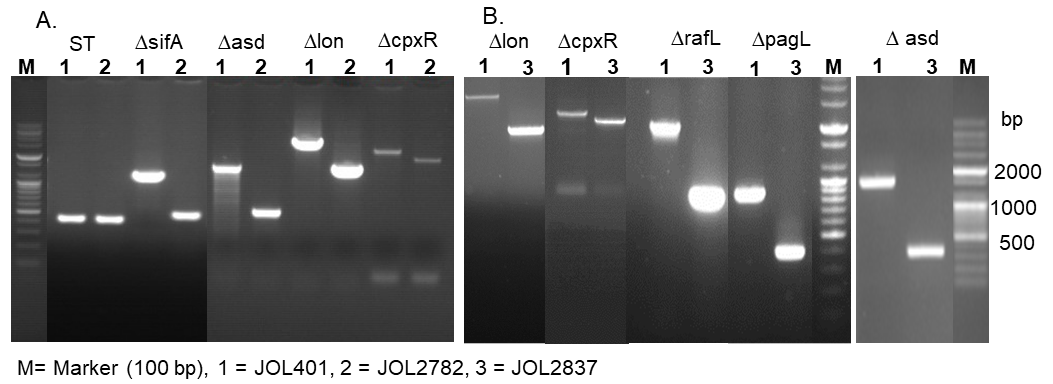

Supplement: Supplementary file 1 — Additional file 1: Confirmation of deletion of genes in Salmonella Typhimurium. The deletion of genes for the engineered Salmonella constructs was confirmed using the respective flanking primers. [file 13567_2022_1096_MOESM1_ESM.tif]
